# Comparative wood anatomy of Korean *Viburnum* L. (Adoxaceae) and its taxonomic implication

**DOI:** 10.3897/phytokeys.156.52031

**Published:** 2020-08-21

**Authors:** Balkrishna Ghimire, Dong Chan Son, Beom Kyun Park, Seung-Hwan Oh

**Affiliations:** 1 Division of Forest Biodiversity, Korea National Arboretum, Pocheon 11186, South Korea Korea National Arboretum Pocheon South Korea

**Keywords:** *
Viburnum
*, wood anatomy, light microscopy, taxonomy

## Abstract

Comparative wood anatomy of *Viburnum* was carried out to understand the differences in wood features amongst the species which might be useful for taxonomic discrimination in the genus. Altogether, nine taxa belonging to five clades were investigated using a sliding microtome and light microscopy. The growth rings are well represented and earlywood and latewood are distinguishable in cross-section. Some of the important wood features include angular, oval and rounded vessels with scalariform perforation plates, opposite to scalariform inter-vessel pitting, rounded pits with slit-like apertures, thick-walled xylem tracheids with simple, rounded bordered pits, diffuse axial parenchyma, uni- and multiseriate rays, 2–4 cells wide. In general, there is a remarkable uniformity in the qualitative wood features in *Viburnum* species, although quantitative measurement showed some disparities. The most significant quantitative wood variables which might be useful for taxonomic groupings of the species comprise a frequency of vessels and rays, the diameter of the vessels and tracheids in the radial and tangential planes and height and width of rays in the tangential plane.

## Introduction

Investigation of wood anatomical features and their correlation with one another, as well as with different habitats and environmental parameters, has been a long practice (Frost 1930, 1931; Carlquist 1975, 2001; Bissing 1982; Metcalfe and Chalk 1983; Baas 1986; Baas and Schweingruber 1987). Plant taxonomists understand that wood anatomical features can provide useful information to unravel the phylogenetic relationships amongst the genera of angiosperms and gymnosperm (Oskolski 1994, 1995; Baas et al. 2000; Oskolski and Lowry 2000; Visscher and Jagels 2003; Lens et al. 2007; Esteban and de Palacios 2009). Nevertheless, due to the insufficient knowledge of the microscopic structure of wood in many taxonomic groups, the studies incorporating wood features in phylogenetic analysis are limited (e.g. Baas et al. 1988; Zhang 1992; Gasson 1994, 1996; Noshiro and Baas 1998; Klaassen 1999; Olson 2002; Malécot et al. 2004; Lens et al. 2007).

The genus *Viburnum* L. (Adoxaceae) consists of about 175 to 230 species of shrubs and small trees distributed in the temperate region of the Northern Hemisphere to the subtropical region of Asia and Latin America (Malécot and McNeill 2002; Donoghue et al. 2004; Moura et al. 2015). The genus was initially classified under the family Caprifoliaceae by Linnaeus (1753) which was later given its own family Viburnaceae, including *Sambucus*, by Rafinesque (1820). However, contemporary molecular phylogenetic analysis (e.g. Backlund and Donoghue 1996; Backlund and Bremer 1997; Kim et al. 1999; Olmstead et al. 2000; Bell et al. 2001; Donoghue et al. 2001) suggested that *Viburnum* and *Sambucus* are more closely related to *Adoxa*. As a result, the Angiosperm Phylogeny Group (APG) system recognises both these genera under the family Adoxaceae (APG 2009, 2016). A proposal to conserve the name Viburnaceae was made by Reveal (2008) and accepted by the General Committee (Wilson 2016) and approved by the Nomenclature Section of the Shenzhen International Botanical Congress. As such, currently the name Viburnaceae, is conserved and has priority over the name Adoxaceae. However, because a super-conservation proposal for the name Adoxaceae has still to be assessed by the relevant committees, ICN Rec. 14A.1 recommends following existing usage, as we have done here in using Adoxaceae as the correct name of the family.

In recent decades, *Viburnum* has been extensively studied and much progress has been made in the understanding of its phylogeny (Donoghue et al. 2004; Winkworth and Donoghue 2004, 2005; Clement and Donoghue 2011, 2012; Clement et al. 2014). Despite the uniform flower and fruit morphology, *Viburnum* species are well known for their striking variation in several morphological features, like presence or absence of naked buds, sterile flowers around the margin of the inflorescence, endocarp shape, inflorescence form and leaf morphology, based on which the genus has been subdivided into ten sections (Hara 1983) or in 12 (Winkworth and Donoghue 2005) to 16 clades (Clement et al. 2014).

In Korea, ten taxa of *Viburnum* belonging to five clades have been described (Hong and Im 2003; Kim 2007). Based on the DNA analysis, Choi et al. (2018) distinguished six out of ten Korean species at the species level. Moreover, Choi and Oh (2019) found that Korean *Viburnum* is easily distinguishable, based on their morphological features, like the character of bud, leaf, extra-floral nectary, inflorescence, corolla, fruit and stone. Wood anatomy of *Viburnum* species is scattered in literature and most wood anatomical studies include a limited number of species from restricted geographical areas (Gundersen 1910; IAWA Committee 1989; Ogata 1988; Eom and Chung 1996; Lens et al. 2016). In this study, we present the wood anatomy of nine out of ten Korean *Viburnum* (except *V. koreanum*). The principal objectives of this study are: (i) to provide an overview of wood anatomical variation within *Viburnum* species, (ii) to identify the systematic significance of wood features in *Viburnum* and (iii) to relate the wood anatomical data to DNA and other morphological features within the *Viburnum*.

## Materials and Methods

### Light microscopy

Mature branches were collected from the natural populations. Names of the studied taxa, voucher number and collection sites are presented in Table 1. Collected wood materials were preserved in 50% ethyl alcohol before sectioning. Preserved wood samples were cut into approximately 2 cm-long circular blocks. For microtome sectioning, the samples were prepared depending on the size of wood; cubic pieces were cut (transverse) or split (radial and tangential) from circular blocks. Three such pieces – representing three planes: transverse, radial and tangential – of each sample were prepared. The blocks were preserved in softener solution (glycerine 10 parts/10% aerosol OT 3 parts/distilled water 87 parts) until sectioning and were sectioned according to the standard technique for light microscopy.

**Table 1.** Name of taxa and collection information. KH, Herbarium Korea National Arboretum.

Taxa	Clade	Voucher no. (KH)	Collection site
*Viburnum dilatatum* Thunb.	Succotinus	Paik, 160607-0012	Korean National Arboretum, Gyeonggi-do
*Viburnum erosum* Thunb.	Succotinus	Paik, 160509-001	Uijeongbu Dobongsan, Gyeonggi-do
*Viburnum japonicum* (Thunb.) C.K. Spreng.	Succotinus	Lee, 160519-010	Dogsilsan, Sinan-gun, Gagaedo
*Viburnum wrightii* Miq.	Succotinus	Paik, 160614-0001	Odaesan, Gangneung, Gangwon-do
*Viburnum burejaeticum* Regel & Herd.	Euviburnum	Paik, 160502-001	Pyeongchang-gun, Gangwon-do
*Viburnum carlesii* Hemsl.	Euviburnum	Paik, 160420-001	Samcheok-si, Gangwon-do
Viburnum odoratissimum var. awabuki (K. Koch) Zabel ex Rümpler	Solenotinus	Lee 160616-014	Seogwipo-si, Sioreum, Jeju-do
*Viburnum furcatum* Blume ex Maxim.	Pseudotinus	Lee, 160429-007	Seogwipo-si, 1100 Hill, Jeju-do
*Viburnum opulus* for. *hydrangeoides* (Nakai) Hara	Opulus	SGUB, 160528-001	Cheorwon-gun, Soisan, Gangwon-do

After preparing permanent slides, microscopic observations and wood-feature analyses were carried out under an AXIO Imager A1 light microscope (Carl Zeiss, Germany). We studied several quantitative and qualitative features of the wood structure and pertinent features of them are summarised in Table 2. Observations and measurements of quantitative features of vessels, tracheids and rays were made with a Hirox 3D microscope (Hirox, Japan). Mean values for each feature were calculated from the measurements taken from the same species, but different samples and standard deviations were also calculated. Photographs of the best sections with characteristic features were taken using a digital camera system attached to the light microscope.

**Table 2.** Distinguishing wood features in *Viburnum* species included in this study.

**Growth rings**	Distinct
**Wood**	Diffuse-porous
**Vessels**	Solitary
**Vessel outline**	Angular/oval/rounded
**Perforation plates**	Scalariform
**Scalariform perforation plate bars**	20–40
**Inter-vessel pits**	opposite, scalariform
**Helical thickenings in vessel elements**	Absent or indistinctly present
**Vessel frequency**	40–220
**Septate fibers**	Absent
**Fiber pits**	Common on radial and tangential walls
**Helical thickenings in fiber cells**	Present
**Axial parenchyma**	Diffuse
**Ray width**	1–4 cells
**Ray in radial section**	Procumbent, upright, square marginal
**Ray frequency**	10–58

### Statistical analysis

The biometric data were analysed statistically. For each wood feature, one-factor analysis of variance (ANOVA) was used to examine differences in means amongst the included species. Pearson’s correlation coefficients were used to estimate the relationship amongst the vessel number (VN), vessel diameter in tangential (VDT) and radial (VDR) planes, vessel wall thickness (VW), fibre diameter in radial (TDR) and tangential (TDT) planes, fibre wall thickness (TW), bordered pit (BP), ray number (RN), ray length (RL) and ray wall thickness (RW). All of the statistical analyses were carried out using the SPSS statistical programme (IBM SPSS Statistics for Windows Version 20.0., IBM Corp., Armonk, USA). Principal component analysis (PCA) was also performed to verify whether wood features allowed the grouping of the species by using the statistical programme R (RStudio, Inc., USA).

## Results

**Figure 1.**
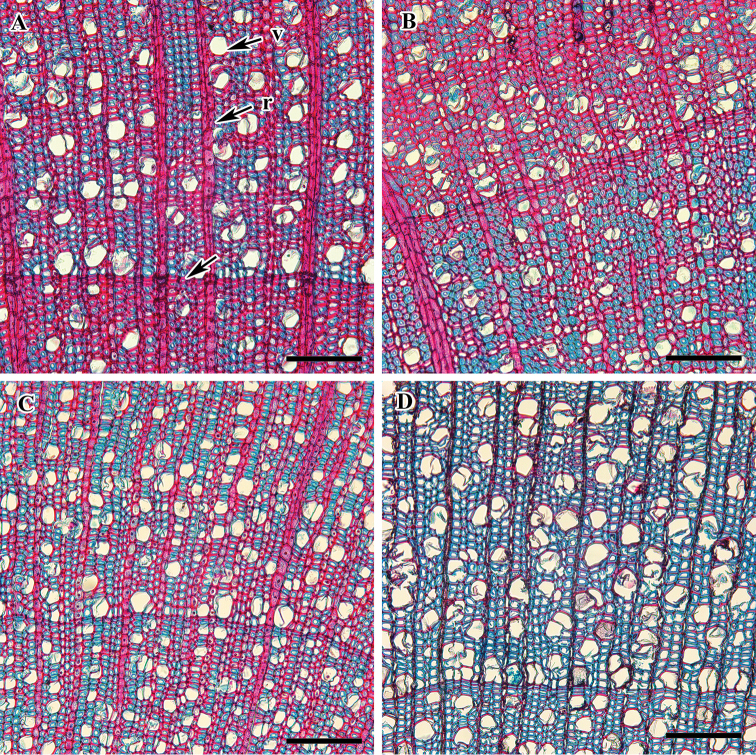
Cross section of *Viburnum* wood showing growth ring, vessels, tracheids, and rays. **A***V. dilatatum* (arrow indicates growth ring) **B***V. erosum***C***V. carlesii***D**V. opulus f. hydrangeoides . Abbreviations: r, ray; v, vessel. Scale bars: 0.1 mm.

**Figure 2.**
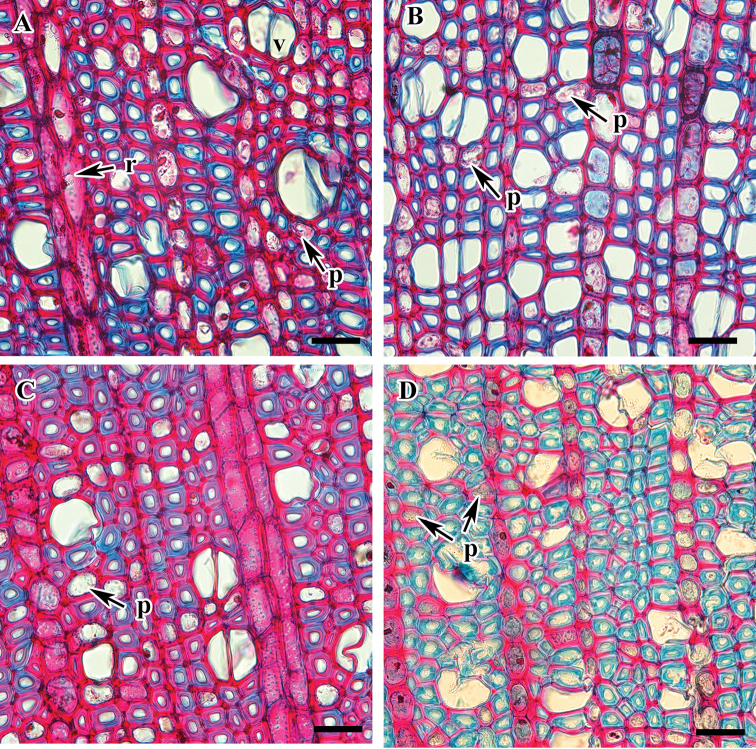
Cross section of *Viburnum* species showing axial parenchyma (magnified). **A***V. japonicum***B**V. ordoratissimum var. awabuki**C***V. wrightii***D***V. furcatum*. Abbreviations: p, parenchyma cell; r, ray. Scale bars: 20 µm.

**Figure 3.**
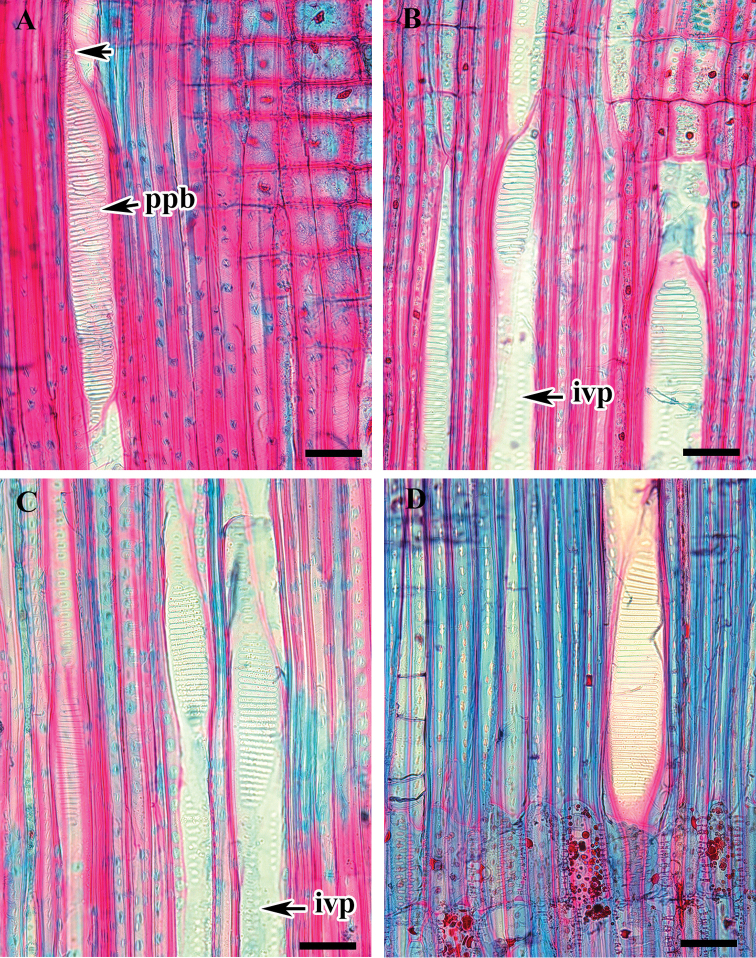
Radial longitudinal section (RLS) of *Viburnum* wood showing inter-vessel pits and scalariform perforation plates. **A***V. wrightii* (arrow indicates gradual vessel tail) **B***V. carlesii***C***V. burejaeticum***D**V. opulus f. hydrangeoides . Abbreviations: ppb, bars on perforation plate; ivp, inter-vessel pits. Scale bars: 20 µm.

**Figure 4.**
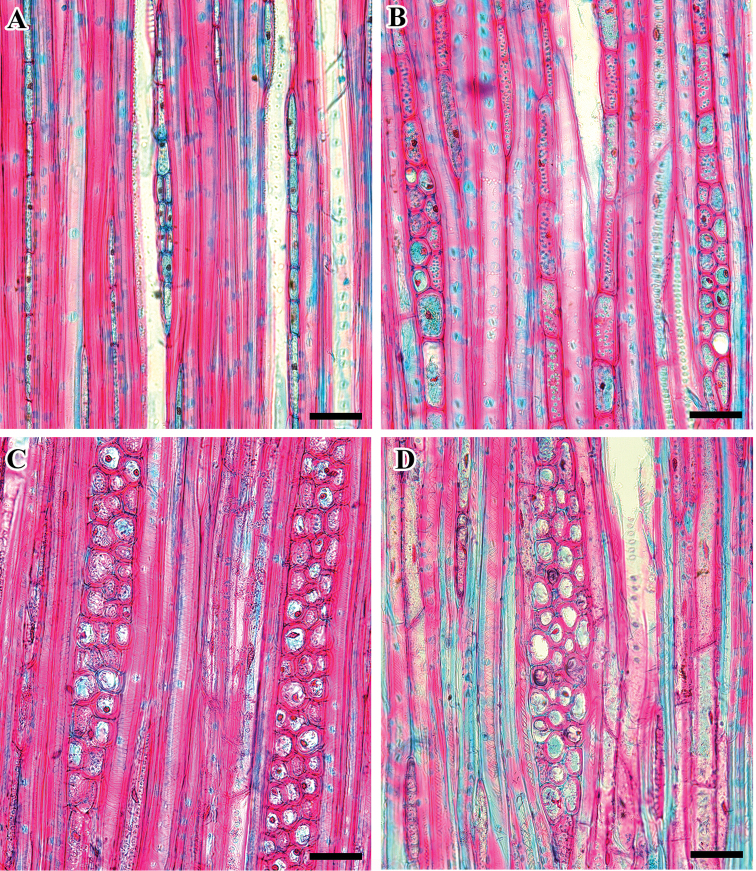
Tangential longitudinal section (TLS) of *Viburnum* wood showing different types of cells in the ray. **A***V. burejaeticum***B***V. carlesii***C***V. dilatatum***D***V. erosum*. Scale bars: 20 µm.

**Figure 5.**
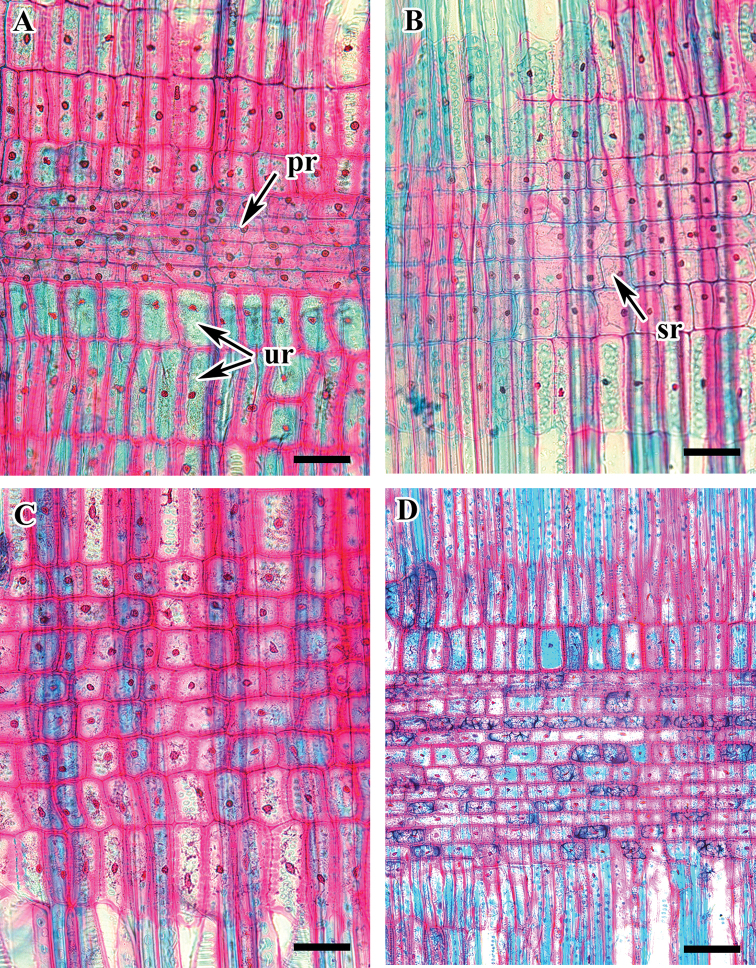
Radial longitudinal section (RLS) of *Viburnum* wood showing ray and parenchyma. **A***V. erosum***B***V. carlesii***C***V. burejaeticum***D***V. furcatum*. Abbreviations: pr, procambium ray cells; sr, square ray cells; ur, upright ray cells. v, vessel. Scale bars: 20 µm.

**Figure 6.**
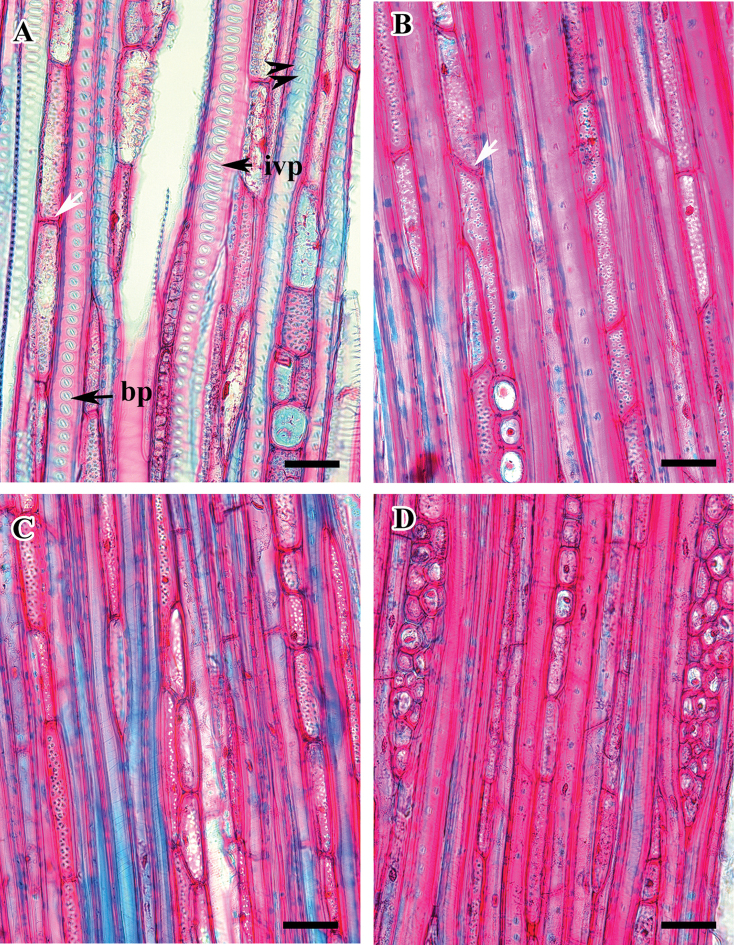
Tangential longitudinal section (TLS) of *Viburnum* wood showing ray and parenchyma. **A***V. wrightii* (double arrow head indicate wall thickenings in tracheids, white arrow indicates simple cross wall in parenchyma cells) **B**V. ordoratissimum var. awabuki (white arrow indicates, oblique cross wall in parenchyma cell) **C***V. japonicum***D***V. dilatatum*. Abbreviations: bp, bordered pits; ivp, inter-vessel pits. Scale bars: 20 µm.

Altogether, nine taxa belonging to five clades of *Viburnum* from Korea were investigated in this study. The qualitative wood features and quantitative wood variables of all included species are presented in Tables 2, 3. Figures 1–6 show the detailed wood features in the cross, radial and tangential sections. All the taxa included have well-defined growth rings with the gradual transition from early to latewood (Figs 1A–D). In all species, the quantity of latewood is very small and the fibres are tangentially elongated with the narrow lumen. The wood is diffuse porous. The vessels are exclusively solitary, narrow, mostly angular, oval and round in cross-section (Figs 1A–D; 2A–D). The widest vessels in radial and tangential diameter are in V. opulus f. hydrangeoides (ranges from 18.8–47.1 µm) and (19.4–44.5 µm), respectively, whereas the narrowest vessels in radial diameter are in *V. furcatum* (11.7–27 µm) and tangential diameter in *V. wrightii* (11.1–25.3 µm). The number of vessels is highest in *V. burejaeticum* (frequency ranges 170–220 mm^-2^) followed by *V. carlesii* (frequency ranges 146–212 mm^-2^) and the lowest number of vessels is recorded in *V. japonicum* (frequency ranges 40–78 mm^-2^) followed by *V. wrightii* (frequency range 56–96 mm^-2^). The vessel wall thickness ranges from 1.6–2.6 µm in *V. burejaeticum* to 2.3–4.2 µm in *V. japonicum*.

**Table 3.** Wood variables in different *Viburnum* species (mean and standard deviation). *Abbreviations*: VN, Number of vessels; VDR, Vessel diameter in radial plane; VDT, Vessel diameter in tangential plane; VW, Vessel wall thickness; TDR, Tracheid diameter in radial plane; TDT, Tracheid diameter in tangential plane; TW, Tracheid wall thickness; BP, Bordered pit; RN, Number of rays; RH, Ray height; RW, Ray thickness.

Taxa	VN (mm^-2^)	VDR (µm)	VDT (µm)	VW (µm)	TDR (µm)	TDT (µm)	TW (µm)	BP (µm)	RN (mm^-2^)	RL (µm)	RW (µm)
V. ordoratissimum var. awabuki	164.06±18.67	23.97±4.57	18.33±3.24	2.43±0.45	8.04±1.65	5.96±1.12	2.65±0.48	6.51±0.72	15.89±2.05	669.6±144	39.14±6.33
*V. dilatatum*	97.83±9.43	28.27±5.53	26.61±4.8	2.77±0.36	6.88±1.45	7.81±1.99	3.55±0.41	6.69±0.76	20.74±2.38	656.11±114.86	34.87±9
*V. erosum*	83.14±10.6	27.98±6.58	25.06±6.38	2.81±0.41	5.65±1.36	6.58±1.67	3.69±0.63	5.53±0.66	19.2±3.23	629.86±81.21	43.11±9.43
*V. japonicum*	53.77±8.64	31.99±5.53	20.46±3.34	3.27±0.47	8.22±2.27	7.89±1.52	3.12±0.64	4.31±0.51	20.77±2.95	680.89±135.11	23.86±4.34
*V. burejaeticum*	195.03±10.06	24.41±7.52	18.55±3.51	2.01±0.29	6.06±1.89	4.7±1.24	2.22±0.36	5.08±1	47.77±5.54	294.53±107.08	9.31±2.53
*V. carlesii*	185.5±17.23	27.05±5.13	20.08±3.64	2.21±0.36	6.79±1.83	6.67±2.19	2.11±0.31	6.64±0.83	44.09±3.97	346.53±94.94	19.93±4.68
*V. furcatum*	112.51±9.78	18.23±3.54	19.14±3.95	2.26±0.37	5.63±1.33	5.15±1.08	2.34±0.39	5.72±0.68	41.23±2.92	479.2±94.38	29.26±4.24
V. opulus f. hydrangeoides	152.06±6.82	35.39±6.81	31.7±6.62	2.61±0.34	8.5±2.43	9.54±3	2.89±0.44	4.59±0.79	36.4±2.93	557.6±104	19.55±4.24
*V. wrightii*	70.17±9.28	26.42±4.16	16.73±3.34	2.33±0.41	7.49±1.76	6.67±1.58	2.97±0.56	4.52±0.74	14.74±2.13	750±170.24	32.53±6.38
**ANOVA**	**F = 683.64 P<0.001**	**F = 26.42 P<0.001**	**F = 42.51 P<0.001**	**F = 34.87 P<0.001**	**F = 12.9 P<0.001**	**F = 24.83 P<0.001**	**F = 47.55 P<0.001**	**F = 55.44 P<0.001**	**F = 564.1 P<0.001**	**F = 63.1 P<0.001**	**F = 109.01 P<0.001**

Perforation plates are scalariform with 20–40 bars and vessel tails are gradual in all species (Fig. 3A–D). Inter-vessel pitting is opposite or scalariform, pits are rounded or oval, with an elliptical or slit-like aperture (Fig. 3B, C). Smooth or faintly helical thickening occurs on the vessel walls. Vessel-ray and vessel axial parenchyma pits are distinctly bordered and are similar to the inter-vessel pits. Tracheids are thick walled (wall thickness ranges 1.6–2.7 µm in *V. carlesii* to 2.4–5µm in *V. erosum*), with helical wall thickenings and narrow lumen. Radial and tangential tracheid diameter ranges (radial: 4–9.1 µm in *V. furcatum* to 4.6–12.7 μm in V. opulus f. hydrangeoides; tangential: 2.9–8.4 µm in *V. burejaeticum* to 5–14.7 µm in V. opulus f. hydrangeoides). The bordered pits are circular with a slit-like aperture and occur in both radial and tangential walls. The diameter of bordered pits ranges from 3.5–5.5 µm in *V. japonicum* to 5.3–8.7 µm in *V. dilatatum*.

The rays are mostly uniseriate rarely biseriate (*V. burejaeticum* and *V. japonicum*), uni- and biseriate (*V. carlesii*, *V. furcatum*, V. opulus f. hydrangeoides and *V. wrightii*), multiseriate, 1–3 seriate (V. ordoratissimum var. awabuki, *V. dilatatum*) or 1–4 seriate (*V. erosum*) (Fig. 4A–D). Three types of cellular composition are found in ray cell: body cells procumbent with mostly 2–4 rows of upright and/or square marginal cells (V. opulus f. hydrangeoides, *V. erosum*), body cells procumbent with more than 4 rows of upright and/or square marginal cells (V. ordoratissimum var. awabuki, *V. carlesii*), body cells square with 2–4 rows of upright marginal cells (*V. furcatum*, *V. burejaeticum*, *V. dilatatum*, *V. wrightii*, *V japonicum*) (Fig. 5A–D). The number of rays per square mm is highest in *V. burejaeticum* (frequency ranges 38–58) followed by *V. carlesii* (ranges 36–51) and lowest in *V. wrightii* (ranges 10–19) followed by V. ordoratissimum var. awabuki (ranges 12–21). The ray height in the tangential section is less than 1 mm for all species. The tallest rays are in *V. wrightii* (ranges 428.3–978.3 µm) followed by V. ordoratissimum var. awabuki (ranges 402.2–912.4 µm), whereas the shortest rays are in *V. burejaeticum* (106–559.3 µm) followed by *V. carlesii* (ranges 134.4–541.9 µm).

Axial parenchyma is diffuse with scanty paratracheal parenchyma in solitary strands adjacent to the vessel elements (Fig. 2A–D). Axial parenchyma consists of tubular cells with mostly oblique or sometimes horizontal end walls (Fig. 6A–D). The wall is smooth with minute pits.

### Statistical analysis

**Figure 7.**
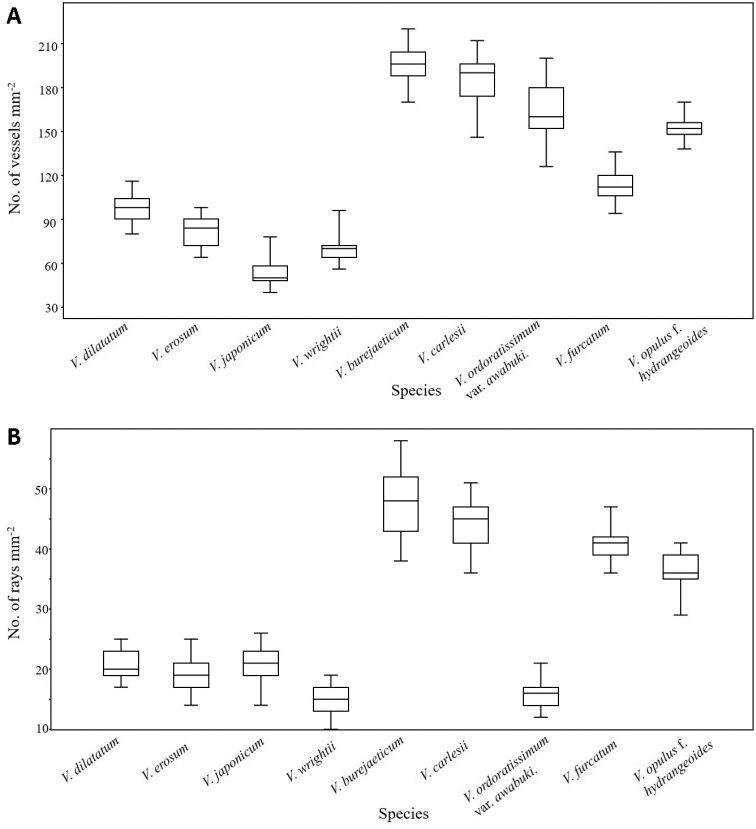
Box plot of ray vessel number and ray numbers per square millimetre in *Viburnum* species.

**Figure 8.**
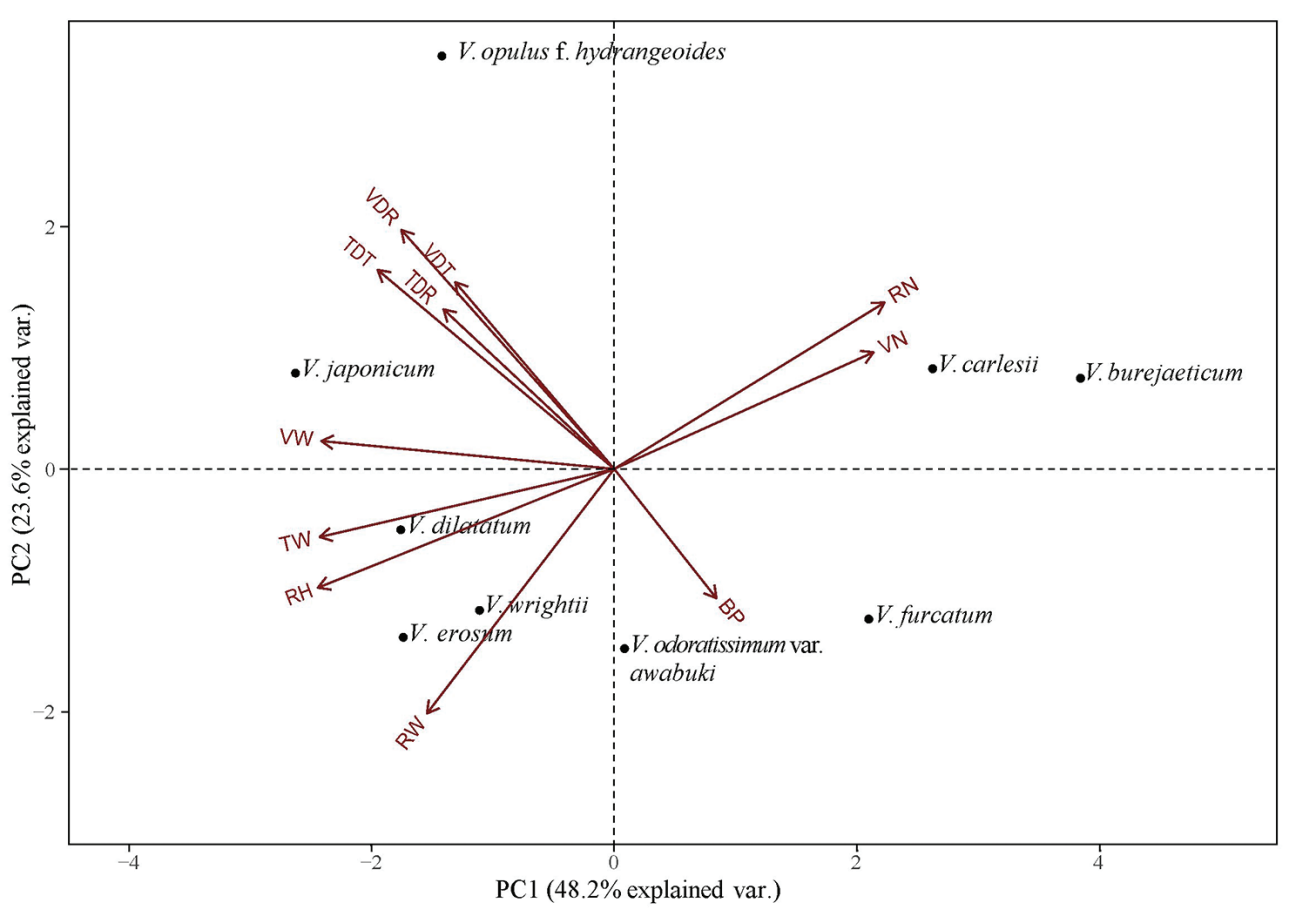
Principal component analysis of 11 different wood variables of *Viburnum* species. VN, Number of vessels; VDR, Vessel diameter in radial plane; VDT, Vessel diameter in tangential plane; VW, Vessel wall thickness; TDR, Tracheid diameter in radial plane; TDT, Tracheid diameter in tangential plane; TW, Tracheid wall thickness; BP, Bordered pit; RN, Number of rays; RH, Ray height; RW, Ray thickness.

One-factor ANOVA was performed on 11 quantitative traits and the differences between species were found to be highly significant (Table 3). Pearson’s correlation coefficient also indicated a significant correlation between wood features (Table 4). Figure 7 (A, B) shows the variation in vessel number and ray number per square millimetre. The relationships amongst the species were revealed using PCA analysis (Fig. 8). The first four components explain 92.61% of the total variance of the analysed data. The first axis of the complete sample set explained 48.21% of the total variance and showed strong positive loadings for vessel numbers and ray numbers per square area (VN and RN) and strong negative loadings for vessel and fibre wall thickness (VW and FW) in association with ray height (RH). The second axis explained 23.61% of the total variance and showed strong positive loadings for vessel and tracheid diameter (VDT, VDR, TDT and TDR) and negative loadings for ray wall thickness and bordered pits (RW and BP). Amongst the four, three species (*V. erosum*, *V. dilatatum* and *V. wrightii*) of the clade Succotinus were grouped on the negative side of both axes whereas *V. japonicum* remained on the positive side of axis two and negative side of axis one. Similarly, both species of the clade Euviburnum, included in this study (*V. carlesii* and *V. burejaeticum*), were grouped on the positive side of both axes.

**Table 4.** Pearson’s correlation coefficients between different wood features in *Viburnum* species. Numbers in bold indicate significant. *Abbreviations*: VN, Number of vessels; VDR, Vessel diameter in radial plane; VDT, Vessel diameter in tangential plane; VW, Vessel wall thickness; TDR, Tracheid diameter in radial plane; TDT, Tracheid diameter in tangential plane; TW, Tracheid wall thickness; BP, Bordered pit; RN, Number of rays; RH, Ray height; RW, Ray thickness.

	VN	VDR	VDT	VW	FDR	FDT	FW	BP	RN	RH	RW
**VN**											
**VDR**	-.109										
**VDT**	-.037	.467^**^									
**VW**	-.496^**^	.278^**^	.274^**^								
**FDR**	-.042	.167^**^	.106	.125^*^							
**FDT**	-.191^**^	.276^**^	.348^**^	.313^**^	.251^**^						
**FW**	-.538^**^	.226^**^	.243^**^	.356^**^	.062	.191^**^					
**BP**	.284^**^	-.160^**^	-.043	-.158^**^	-.125^*^	-.167^**^	-.058				
**RN**	.650^**^	-.129^*^	.006	-.386^**^	-.169^**^	-.115^*^	-.545^**^	.050			
**RH**	-.604^**^	.117^*^	.042	.345^**^	.165^**^	.213^**^	.428^**^	-.128^*^	-.725^**^		
**RW**	-.445^**^	-.068	.055	.253^**^	-.050	.079	.412^**^	.208^**^	-.656^**^	.556^**^	

**Sig. at 0.01 level *Sig. at 0.05 level.

## Discussion

Descriptions of wood anatomical features of some *Viburnum* species from different geographical areas are available in literature (Gundersen 1910; Ogata 1988; IAWA Committee 1989; Eom and Chung 1996; Lens et al. 2016). Lens et al. (2016) confirmed that the wood anatomy of *Viburnum* perfectly fits within the Dipsacales and also showed some key differences with its close relative *Sambucus*. In his report, the wood anatomy of the Caprifoliaceae of Japan, Ogata (1988) included 13 species of Japanese *Viburnum* along with *Sambucus racemosa* and compared with other members of the Caprifoliaceae (*Abelia*, *Lonicera*, *Weigela* and *Zabelia*). In this early 21^st^ century, phylogenetic relationships, based on molecular data, imply revision of familial limits in Dipsacales and APG (2009, 2016) classification included both *Viburnum* and *Sambucus* in Adoxaceae family, which is sister to Caprifoliaceae. In this study, we compared the wood features of nine out of ten species of *Viburnum* distributed in Korea.

### Relationship amongst wood variables

Most of the characteristic wood features observed in this study, for instance, distinct growth rings, diffuse porous wood, solitary vessel, scalariform perforation plates, opposite or scalariform inter-vessel pits, wall thickenings in tracheids, diffuse axial parenchyma and heterocellular rays are similar to those reported in previous studies on *Viburnum* (Ogata 1988; IAWA Committee 1989; Lens et al. 2016). However, we found variations in quantitative traits, such as vessel density, vessel diameter, ray width, ray height and ray density. When compared, the relatively lower numbers of vessels per square millimetre were counted for some species in our samples particularly, *V. carlesii* (146–212 vessels/mm^2^) and *V. erosum* (64–98 vessels/mm^2^) than that of Ogata (1988) who observed 168–294 and 90–117 vessels/mm^2^ for these species, respectively. On the other hand, we recorded slightly higher numbers of vessel/mm^2^ for *V. furcatum* (94–136 vessels/mm^2^) than Ogata (1988) (58–128 vessels/mm^2^) and for *V. dilatatum* than Lens et al. (2016) (60–82 vessels/mm^2^). Our result was more comparable with Lens et al. (2016) for *V. carlesii* (150–185 vessels/mm^2^) and *V. furcatum* (100–130 vessels/mm^2^) in terms of vessel frequency, but they did not observe *V. erosum*. However, for the rest of the species, we found fairly similar data to Ogata (1988), excluding V. ordoratissimum var. awabuki, *V. burejaeticum* and V. opulus f. hydrangeoides which were not included on his observations.

The ray height (RH) barely exceeds one millimetre in all the taxa included in this study. However, Ogata (1988) recorded rays more than one millimetre (even 4+ mm in *V. furcatum*) in height for all species he studied. Young (2015) also found rays more than one millimetre high in V. ordoratissimum var. awabuki. Although the heights of the tallest rays were over one millimetre, the average ray heights recorded by Lens et al. (2016) for *V. dilatatum*, *V. carlesii* and *V. furcatum* are similar to ours. The observations were also similar for ray width. The widest rays were in *V. dilatatum* and *V. erosum* with one- to three- and four- celled wide rays, respectively, whereas the remaining species usually had one- to two- (rarely three- in V. ordoratissimum var. awabuki) celled rays.

In general, we found 10–19 (*V. wrightii*) to 38–58 (*V. burejaeticum*) rays per square millimetre. Although we did not count multi- and uni-seriate rays separately, these numbers are higher than those of Lens et al. (2016) as they counted 6–9 multiseriate and 7–9 uni-seriate rays in *V. carlesii* which has the highest number of rays per square millimetre in their observation. On our side, we found 36–51 rays in *V. carlesii* which is the second-highest number in our observations. The result of ANOVA indicated that the variation in the ray number (RN) in the *Viburnum* species is significant (*P* < 0.001) (Table 4). In addition, there was a strong negative significant relationship between ray numbers and ray height (RL) (*r* = -0.725; *P* < 0.001) and ray width (RW) (*r* = -0.656; *P* < 0.001) and a strong positive relationship between ray number and vessel number (*r* = -065; *P* < 0.001) (Table 4).

The analysis of biometric data indicated that vessel wall thickness (VW) is positively related to vessel diameter in both planes (VDR and VDT) and negatively related to vessel numbers per square millimetre. It is noteworthy that the vessel number and vessel diameter in both planes are also negatively related, but without statistical significance. This is an obvious and well-known phenomenon related to the water transport system according to Baas (1973) and Carlquist (1975). Wider vessels are more efficient water conductors than narrow ones, but they are more vulnerable against cavitation (Baas et al. 1983; Choat et al. 2005).

### Taxonomic significance of wood variables

We included nine taxa belonging to five clades of *Viburnum* for the comparative study. The phylogeny of *Viburnum* is very well studied using DNA of both nuclear and chloroplast regions incorporating some morphological features (Donoghue et al. 2004; Winkworth and Donoghue 2004, 2005; Clement and Donoghue 2011, 2012; Clement et al. 2014). However, we have not found any reports of *Viburnum* wood structures considered as a diagnostic feature for the genus as a whole. According to Lens et al. (2016), scalariform perforations in *Viburnum* is the only wood feature that distinguished this genus from its close relative *Sambucus* which has simple perforations. Although our study did not find any particular wood features that support intraspecific relationship within the genus, biometric data exhibited some groupings which are almost congruent with current phylogeny of *Viburnum*. In particular, our PCA result revealed groups that corresponded closely to the clades identified by molecular analyses (Clement et al. 2014; Choi et al. 2018).

*Viburnum dilatatum*, *V. erosum*, *V. wrightii* and *V. japonicum*, members of clade Succotinus, formed a highly-supported clade in the phylogenetic tree inferred from chloroplast and nuclear DNA (Choi et al. 2018). Morphologically, these species are characterised by free bud scales, serrate leaves with pinnate veins and an extra-floral nectary at proximal regions on the abaxial leaf surface (Choi and Oh 2019). Our results partially agree with previous studies, as these species exhibited comparable wood variables, for instance; vessel and ray numbers per square millimetre (Fig. 7A, B), vessel diameter, tracheid wall thickness and ray height and thus form a close group in the PCA plot (Fig. 8). A similar result is obtained for *V. carlesii* and *V. burejaeticum* which belongs to clade Euviburnum. These two species are grouped together on the positive axis of both components and exhibit comparable vessel and ray numbers per area, vessel and tracheid diameter and wall thickness. These two species with stellate trichomes on branchlets, petioles and leaves, naked buds and stones with two dorsal and ventral grooves also form strongly-supported monophyletic groups in a phylogenetic analysis (Choi et al. 2018; Choi and Oh 2019).

In conclusion, despite the limited taxa sampling from a restricted geographical region, the results of this study demonstrated the considerable quantitative variations that exist in the wood features of *Viburnum* species. Although most of the qualitative wood features exhibited uniformity amongst the species, quantitative variables displayed significant relationships with each other and also provided some support for the taxonomic groupings. Further studies considering many more species from different clades will help to clarify the taxonomic problems within the genus. In addition, the methods used in this study can be applied to other taxa, as well as offering valuable basic information about how the wood variables can contribute to taxonomic relationships.
